# Impact of Semantic Relatedness on Associative Memory: An ERP Study

**DOI:** 10.3389/fnhum.2017.00335

**Published:** 2017-06-29

**Authors:** Pierre Desaunay, Patrice Clochon, Franck Doidy, Anna Lambrechts, Dermot M. Bowler, Priscille Gérardin, Jean-Marc Baleyte, Francis Eustache, Bérengère Guillery-Girard

**Affiliations:** ^1^Normandie Univ, UNICAEN, PSL Research University, EPHE, INSERM, U1077, CHU de Caen, Neuropsychologie et Imagerie de la Mémoire HumaineCaen, France; ^2^Fédération Hospitalo-Universitaire de Psychiatrie de l’Enfant et de l’AdolescentRouen, France; ^3^Department of Psychology, City, University of LondonLondon, United Kingdom; ^4^Service de Psychiatrie de l’enfant et de l’adolescent, Centre Hospitalier Intercommunal de CréteilCréteil, France

**Keywords:** episodic memory, ERP, old/new effect, binding, pictures

## Abstract

Encoding and retrieval processes in memory for pairs of pictures are thought to be influenced by inter-item similarity and by features of individual items. Using Event-Related Potentials (ERP), we aimed to identify how these processes impact on both the early mid-frontal FN400 and the Late Positive Component (LPC) potentials during associative retrieval of pictures. Twenty young adults undertook a sham task, using an incidental encoding of semantically related and unrelated pairs of drawings. At test, we conducted a recognition task in which participants were asked to identify target identical pairs of pictures, which could be semantically related or unrelated, among new and rearranged pairs. We observed semantic (related and unrelated pairs) and condition effects (old, rearranged and new pairs) on the early mid-frontal potential. First, a lower amplitude was shown for identical and rearranged semantically related pairs, which might reflect a retrieval process driven by semantic cues. Second, among semantically unrelated pairs, we found a larger negativity for identical pairs, compared to rearranged and new ones, suggesting additional retrieval processing that focuses on associative information. We also observed an LPC old/new effect with a mid-parietal and a right occipito-parietal topography for semantically related and unrelated old pairs, demonstrating a recollection phenomenon irrespective of the degree of association. These findings suggest that associative recognition using visual stimuli begins at early stages of retrieval, and differs according to the degree of semantic relatedness among items. However, either strategy may ultimately lead to recollection processes.

## Introduction

Episodic memory refers to the most complex human memory system and requires both the individual’s self-awareness (i.e., autonoetic consciousness) of having personally experienced a past event while retrieving the overall phenomenological details (i.e., context or source) bound to that unique moment (which gives a peculiar vividness to the recall) and the ability to make sense of this recall for future experiences (Tulving, 2002; Eustache and Desgranges, 2008). Thus, episodic memory processes rely on the binding of different pieces of information into a coherent event. In that sense, associative memory paradigms are well suited to test the ability to bind two or more items into a unique mental representation. These tasks involve memorizing each item (item 1, item 2) as well as the associative information (item1–item 2) and may lead to the use of different strategies based either on similarities between items or on item-specific features. The existence of a semantic association would favor a processing of common properties (global matching model, for review see Clark and Gronlund, 1996), based on pre-existing associations (Wang and Morris, 2010). The encoding of related items is driven both by common visual similarity as well as by conceptual similarity (for review see Buchler et al., 2008), and attentional processes allow self-initiated semantic encoding, extracting commonalities between pairs of items (i.e., semantic information), which provide retrieval cues in the test phase (Hawco et al., 2013). Alternatively, when there is no semantic association between items, recall of weakly associated items places greater reliance on episodic, contextual information and the consequent specificity of retrieval cues (Mäantylä, 1986). Experimental and neuroimaging data confirmed that recollection as well as familiarity are implicated in associative memory (Addante et al., 2012; Wang et al., 2012). However, they are differentially affected by the nature of any association between two studied items (Yonelinas, 2002).

These effects of semantic relatedness on recollection and familiarity processes were also investigated using Event-Related Potentials (ERP). ERP, thanks to its high temporal resolution, enables the exploration of temporal profile of the cognitive processes implicated in associative recognition. ERP analyses enable us to separate processes such as familiarity and recollection based upon their temporal effects. They have identified two successive but independent old/new effects, consisting of a greater positivity for correctly recognized old as compared to correctly rejected new items, successively on the mid-frontal negative potential FN400 (300–500 ms) for familiarity, and the Late Positive Component (LPC; 500–800 ms) for recollection (for review see Friedman and Johnson, 2000). Moreover, the specific sensitivity of ERP to the manipulation of experimental factors enables us to tease out their implications for different perceptual and cognitive processes. Thus ERP studies provide information that can further illuminate models of associative memory. Using word pairs, a growing corpus of EEG studies have confirmed that familiarity can support associative recognition when the to-be-associated stimuli are unitized into a single and coherent representation (for review see Zimmer and Ecker, 2010).

However, using verbal material limits the study the role of perceptual information in associative memory. Recently, a study of associative recognition using pairs of fractals identified three successive old/new effects: early visual P100 (100–175 ms) responsive to novelty, then mid-frontal (300–500 ms), then late parietal (500–800 ms), suggesting that associative memory for visual material is a combination of perceptual priming, familiarity and recollection (Speer and Curran, 2007). More recently, Tibon et al. (2014b) used pairs of pictures of objects that were either semantically related or unrelated. During the associative recognition phase, they identified a FN400 old/new effect for related pairs only, suggesting that the pictures could be unitized semantically. In that study, participants were instructed to remember object-picture pairs presented in a vertical configuration and close to each other (a scoop of ice cream above an ice cream cone), and to perform two judgments: to choose the object they preferred and to decide which of the two objects was more common. Hence, the semantic link was driven by the functional configuration of the items, with the process of unitization being manipulated by the pairs being presented twice or three times. Another study published by the same author used cued recall to test retrieval of associative information between unrelated pairs of pictures (Tibon and Levy, 2014a). Picture cues were presented at test, and subjects were asked to recall the name of the associated target pictures. Unsuccessful recall was associated with a more negative FN400 during the presentation of the cue, which the authors argued was a reflection of working-with-memory operations (Moscovitch, 1992) such as frontal mechanisms tapping medial temporal lobe representations until recollection begins. As a result, FN400 waveforms seem sensitive to encoding operations occurring during associative retrieval. Old/new effects, reflecting familiarity-based recognition, have been found when encoding conditions favor unitization in particular with semantically related items (Rhodes and Donaldson, 2007). However, very few studies have examined ERPs when semantic link is used as the main associative recognition cue.

The aim of the present study is to identify how the semantic relatedness as a retrieval cue, would modify both the early mid-frontal FN400 and the LPC potentials during associative recognition of pictures. Pictures have both visual and verbal codes (dual-coding hypothesis, Paivio, 1971), that implicate complementary perceptual and semantic processes. We used colored line drawings as stimuli, to reduce the perceptual complexity of stimuli and highlight both the within-pair similarity and the specific features of items, and yield more vivid mental representations. Unlike Tibon et al. (2014b) study, where functional links were highlighted by the vertical arrangement of the pictures in the studied pairs to foster unitization, here, we presented pairs in a horizontal configuration, one at a time, in order to focus attention on semantic rather than functional links. Participants were instructed to imagine a situation that combined both pictures, to favor a more naturalistic context that yielded local and global processing.

On behavioral level, we hypothesized that semantic associations would foster associative recognition but increase false alarms, and on the contrary distinctiveness of non-related pairs would favor a better discrimination between identical and rearranged pairs. We predicted both a better recognition score for related pairs and fewer false alarms for unrelated ones. For ERP results, our working hypotheses are: (1) on the FN400 potential related and unrelated pairs would differ due to semantic effect, we predict that semantic processing of related pairs would modulate the FN400 waveform, associated with a possible but uncertain old/new effect because the study is not designed to favor unitization; and (2) on the LPC, in accordance with dual-process theory, associative retrieval would rely on recollection, with a larger LPC old/new effect for unrelated pairs due to their specificity.

## Materials and Methods

### Participants

Thirty three healthy adults participated in this study. Thirteen participants were excluded due to EEG constraints: low behavioral accuracy (*n* = 7), or too many EEG artifacts arising from ocular movements, large alpha waveforms, or interfering heart rate signal (*n* = 6). As a result behavioral and ERP analyses were run on a total of 20 healthy participants aged from 20 to 32: 9 male, mean age 24.8 ± 3.33 years old (age range 20–32 years), mean level of education 5.25 ± 1.92. (in years of post-primary education), right-handed scoring 11 ± 1 on the Edinburgh Handedness Inventory (Oldfield, 1971). Criteria for exclusion were a diagnosis of mental disorder or mental retardation, or use of psychotropic medication. This study constitutes the first part of an extended project on associative memory in Austim Spectrum Disorder approved by the local Ethics Committee (CPP Nord Ouest III, N° ID RCB : 2013-A01800-45). We obtained written informed consent from each participant after a comprehensive description of the study.

### Stimuli

Four hundred colored line drawings were used, depicting either objects or animals drawn from 20 semantic categories (Supplementary Data, list of semantic categories), each category comprising 20 items. Semantically related pairs were created by pairing pictures within each semantic category, and unrelated pairs consisted in paring two pictures from different categories. Eighteen categories were selected from Marchal and Nicolas (2003), and for each category the 20 first distinct items generated were retained. A 19th (jewels) and a 20th (prepared food) category were generated based on a Wikipedia search. All stimuli were drawn to the same size (not scaled) on a same-color background in 300 × 300 px squares. Within the same semantic category half of the drawings were presented on the left, and the other half on the right.

### Experimental Design (Figures 1, 2A)

At study, 80 semantically related pairs (e.g., piano-trumpet) and 80 semantically unrelated pairs (e.g., watering can-cap) were presented. Within related pairs, we avoided semantic associations (i.e., semantically related items with highest typicality), and intra-categorical associations (e.g., pairing “stringed instruments” together within the category “musical instruments”). Within unrelated pairs, we avoided supra-categorical pairings (e.g., pairing “pets” and “wild mammals”), and functionally associated items pairings (e.g., nail-hammer), in order to avoid unitization. At test, 40 semantically related pairs and 40 semantically unrelated pairs were presented exactly as during the learning phase (“related identical” and “unrelated identical” pairs). In order to differentiate item memory and relational memory, the remaining 40 semantically related and 40 semantically unrelated pairs were rearranged (“related rearranged” and “unrelated rearranged” pairs). To test the classic old/new effect, 40 semantically unrelated new pairs were also presented. In order to control for a purely perceptual association between paired items and a relational association between items, the position of half the identical and rearranged pairs was swapped during the test phase.

**Figure 1:**
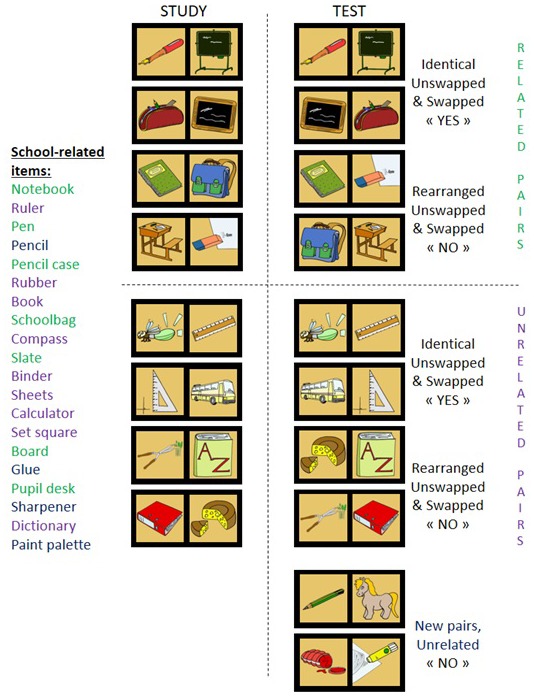
Each of 20 semantic categories contains 20 pictures (e.g., furniture on the left). At study phase, eight items (green) are paired to create strongly related pairs, eight others (purple) are intermixed with items from other categories to create unrelated pairs, while the remaining four (blue) are intermixed with items from other categories and used to create new pairs presented only at test. For the test phase, half of strongly related pairs used at study remained similar (identical related pairs), while other half was rearranged (rearranged related pairs). The same applies to unrelated pairs. Positions of items within a pair were swapped for half identical and rearranged pairs.

**Figure 2:**
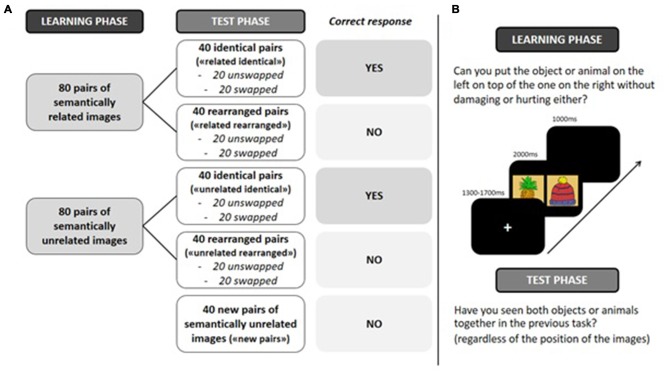
**(A)** Experimental design. **(B)** Experimental procedure. “related identical”: semantically related identical pairs; “related rearranged”: semantically related rearranged pairs; “unrelated identical”: semantically unrelated identical pairs; “unrelated rearranged”: semantically unrelated rearranged pairs; “new pairs”: semantically unrelated new pairs.

The semantic association for each pair was tested with 20 healthy adults (four males, mean age 21.7 ± 2.85 years) different from the 20 previous participants. For semantically related pairs, the semantic link was identified in 97.67% of cases, whereas for unrelated ones no semantic link was identified in 97.90% of cases.

### Procedure (Figure 2B)

Stimulus presentation was controlled by Eprime Pro on a 17″ LCD screen with a 1280 × 1024 resolution. To ensure both that stimuli were not perceived as a single image but still fit in the visual field to avoid exploratory eye movements, pictures of a pair, each one measuring 300 × 300 px squares, are 60 px squares apart (i.e., distant of 30 px squares from the fixation cross). Participants were seated comfortably in a dimly lit room during the whole experiment at a distance from 90 cm to 100 cm from the screen and were instructed to minimize blinking and moving during recording. In both study and test phases, a trial started with a white fixation cross presented on a black background for a pseudo-random interval of 1300–1700 ms, followed by a pair of images (presented in pseudo-random order) for 2000 ms, and next a blank screen for 1000 ms. During the incidental learning phase, in order to enhance a deep and relational encoding, participants were instructed to imagine putting the object/animal on the left onto the object/animal on the right, and to decide whether this would be possible in reality without damaging or hurting either the object or the animal. During the surprise recognition phase, participants were instructed to indicate whether they had seen both images together during the learning phase (old) or not (new), regardless of the position of the images on the screen. Participants were instructed to respond as quickly as possible on one of two keys on a response box. Both phases were preceded by a training phase using five mock items. The study phase lasted 15 min, and the test phase 12 min. An interval of 15 min separated the learning phase and the test phase.

### EEG Recording

EEG activity was recorded continuously by GES 300 amplifier (Electrical Geodesics, INC.) using an EGI Hydrocel Geodesic Sensor Net (HGSN-128) with dense array of 128 Ag/AgCl sensors (Tucker, 1993). Impedances were kept under 100 kΩ (Ferree et al., 2001), and EEG channel was referenced to a vertex reference Cz and ground to CPPZ (fixed by the EGI system). The signal was sampled at 20 kHz frequency with a 24-bit A/D and was online (hardware) amplified and low pass filtered at 4 KHz. However, NetStation software cannot currently acquire at any rate faster than 1 KHz. Hence, the signal was filtered by a Butterworth low pass Finit Inpluse Response (FIR) filter at 500 Hz and sub-sampled at 1 KHz. Electro-oculogram was recorded using four electrodes placed vertically and horizontally around the eyes. Before exporting EEG data, given that we use our own EEG processing software developed in-laboratory and since the amplifier are a DC coupled amplifier, EEG data were processed offline using Netstation 4.4.2 (Electrical Geodesics Inc., Eugene, OR, USA). The signal was filtered using a 1 Hz Kaiser FIR first order high-pass filter (which ensures a linear phase and no distortion in the bandwidth) in order to discard DC and very slow waves. Recordings were re-referenced offline to a common average reference (Bertrand et al., 1985; Tucker et al., 1994) to minimize the effects of reference-site activity and accurately estimate the scalp topography of the measured electrical fields (Dien, 1998). The artifact in EEG stimulus signal was excluded of the analysis by visual inspection. No other corrections and electrodes reconstructions were applied. ERP waveforms were created by averaging the ERPs and the signal was segmented into stimuli-synchronized epochs, which were extracted at 250 ms before (baseline) and until 1000 ms post stimulus onset. Trials were discarded from subjects for whom every individual response conditionalized. ERP average did not comprise at least 15 artifact-free trials (number of trials for “new pairs”: 15–30; “unrelated identical”: 15–22; “unrelated rearranged”: 15–23; “related identical”: 16–22; “related rearranged”: 15–24; Supplementary Table S2). Finally, the evoked potential was then baseline-corrected.

### Debriefing

Immediately after the EEG recording, we administered a 7-question debriefing questionnaire. Each participant was asked: (1) if he noticed semantically related pairs; (2) in which proportion; (3) to name the observed semantic categories spontaneously; or (4) helped by a cue; (5) if the semantic link was helpful or not at study; and (6) at test; and (7) if swapping the position of pictures within pairs was helpful or not. Participant could give several answers to questions 6 and 7.

### Analysis

#### Behavioral

Analysis of behavioral data was conducted using IBM SPSS version 21. We measured accuracy (proportion of correct responses in each condition), and calculated the associative discrimination index Pr (percentage of hits minus percentage of false alarms, Snodgrass and Corwin, 1988). We ran analysis of variance (ANOVAs) using a General Linear Model procedure. *Post hoc* multiple comparisons were Tukey-corrected.

#### Event-Related Potentials (ERP)

The ERP analysis was run on the 20 participants, using SAS software (SAS Institute Software). We included only those participants whose performance provided a minimum of 15 exploitable epochs for each type of trial (suitable epochs had to be associated with both a correct answer and a clean EEG signal).

We used *a priori* defined latencies of interest according to the literature and confirmed by visual inspection in ERP grand-average. For FN400 effect in Cz (electrodes FFC1h, FFC2h, FCC3h, FCC1h, FCZ, FCC4h, FCC2h), we focused the analysis on 400–600 ms latency, based upon literature using single visual items (FN400 peaking near 450 ms—Berman et al., 1990; Noldy et al., 1990; Armilio, 1997; Maher and Stephen, 2008), or visual complex and associative information (Tibon et al., 2014b), as well as complex paradigms requiring associative and source information (FN400 starting at 400 ms—Addante et al., 2012). In our experiment, the FN400 effect occurred during the time window 420–500 ms, and was immediately followed by the late parietal (LPC) effect in the 500–600 ms time window. Using pictures in associative memory, the FN400 time window can also fall in the later part of the range of the ERP, due to the increased demands of associative information or the complexity of these stimuli (Tibon et al., 2014b). Studies usually report that particular LPC as lasting longer, between 400 ms and 500 ms (Johnson et al., 1998; Friedman and Johnson, 2000; Vilberg et al., 2006; Woodruff et al., 2006), but shorter time-windows such as 200 ms duration are also described in studies using pictures with single item (450–700 ms, Ally and Budson, 2007) or associative (Tibon et al., 2014b) recognition, or when high semantic relatedness between pictures (450–600 ms; Paz-Caballero et al., 2006) as well as words (550–650 ms, Molinaro et al., 2012).

In order to focus on associative processes and to have a sufficient number of trials per condition, data for unswapped and swapped pairs were therefore collapsed across each type of trial. According to Speer and Curran (2007), varying the position of visual stimuli within a pair from one trial to the next has no effect on the FN400 and the LPC old/new effect. For the LPC analysis, electrode sites for analysis included CPz (electrodes CPZ, CCP2h, CPP1h, P1, PZ, P2) and were extended to parietal areas (electrodes CP2, CP4, CP6, TP8, P6, PO4, P8) and occipital Oz (electrodes POO1, POZ, OZ, POO2) areas, based on ERP literature with visual stimuli (Achim and Lepage, 2005).

## Results

### Behavioral Data

#### Accuracy (Figure 3)

All accuracy results were higher than 0.50 (Supplementary Table S1), with chance level (proportion of correct answers produced by guessing) equal to 0.40 (i.e., the two hits conditions/a total of five conditions). A 2 (condition: identical, rearranged) × 2 (semantic: semantically related, semantically unrelated) ANOVA revealed a main effect of condition (*F*_(1,20)_ = 8.98, *p* = 0.004; $\eta_{\text{p}}^{\text{2}}$ = 0.11) and a main effect of semantic (*F*_(1,20)_ = 6.11, *p* < 0.01; $\eta_{\text{p}}^{\text{2}}$ = 0.07). Participants were better at rejecting rearranged pairs (Acc_rearranged pairs_ = 0.68) than identifying identical pairs (Acc_identical pairs_ = 0.62), and showed higher accuracy for semantically unrelated pairs (Acc_semantically unrelated_ = 0.67) than for semantically related pairs (Acc_semantically related_ = 0.62). The analysis also revealed a significant condition × semantic interaction (*F*_(1,20)_ = 11.99, *p* = 0.001; $\eta_{\text{p}}^{\text{2}}$ = 0.14). *Post hoc* paired *t*-tests indicated a higher accuracy for “unrelated rearranged” than any other type of pair (all *p*s ≤ 0.0001). We did not find any difference between “related identical” pairs (Acc_“related identical”_ = 0.62) and “unrelated identical” pairs (Acc_“unrelated identical”_ = 0.60, *p* = 0.84). The discrimination index for “unrelated identical” pairs was 0.42 (SD = 0.11) and for “related identical” pairs was 0.32 (SD = 0.11). A one-way ANOVA (*F*_(1,20)_ = 8.25, *p* = 0.0066; $\eta_{\text{p}}^{\text{2}}$ = 0.17) revealed this difference to be significant.

**Figure 3:**
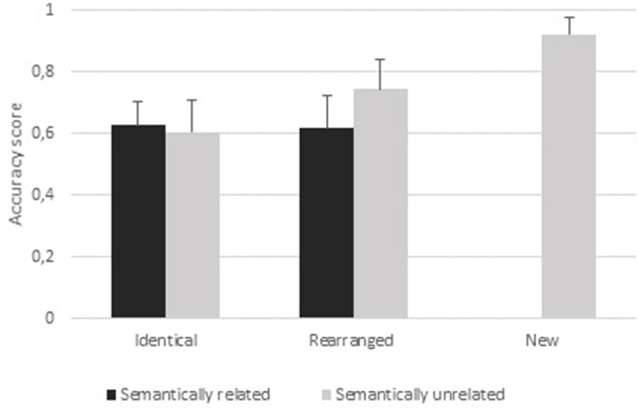
Behavioral results. Accuracy scores per condition (identical pairs, rearranged pairs, new pairs), semantic relation (related, unrelated); errors bars represent standard deviations.

### ERP

#### FN400: Familiarity

##### Old/new effect (Figure 4A)

We conducted paired *t*-tests for all conditions to estimate old-new effects: “related identical”, “related rearranged”, “unrelated identical”, “unrelated rearranged” vs. new pairs. The comparison between “unrelated identical” vs. “new” conditions indicated that the amplitude of FN400 was larger for “unrelated identical” pairs than for new pairs (*t*_(38)_ = −2.62; *p* = 0.01); there was no other significant difference (*p* = 0.35 to *p* = 0.83). This seems to indicate that a larger FN400 is associated with the retrieval of remembered associative information, independently from item familiarity. We have tested the old/rearranged effect by comparing “unrelated identical” with “unrelated rearranged pairs and observed that the amplitude of “unrelated identical” was greater than “unrelated rearranged pairs” (*p* = 0.009).

**Figure 4:**
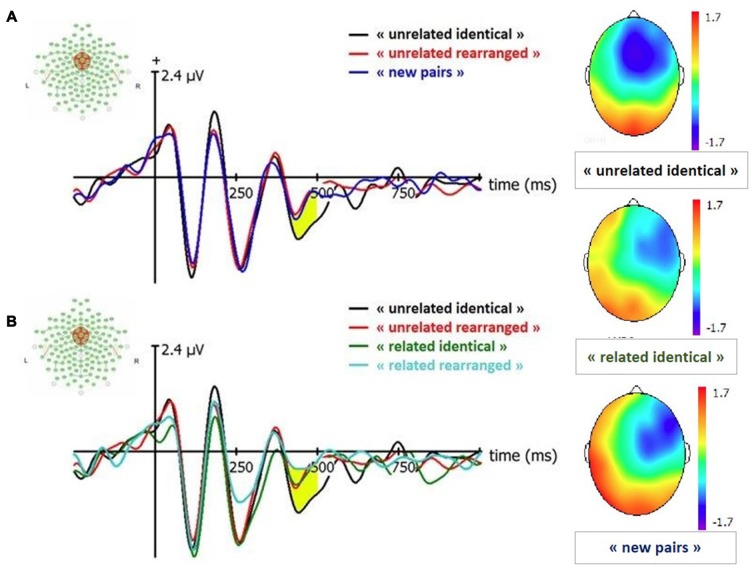
Event-Related Potentials (ERP) and topographies for FN400 (signal in Cz between 420–500 ms). “related identical”: semantically related identical pairs; “related rearranged”: semantically related rearranged pairs; “unrelated identical”: semantically unrelated identical pairs; “unrelated rearranged”: semantically unrelated rearranged pairs; “new pairs”: semantically unrelated new pairs. **(A)** Old/New effect for unrelated pairs. **(B)** Effect of semantic factor on Condition. Yellow shaded areas correspond to significant differences between conditions.

##### Effect of semantic relatedness (Figure 4B)

A 2 (condition: identical, rearranged) × 2 (semantic: semantically related, semantically unrelated) ANOVA conducted on the amplitude of the FN400 revealed a significant effect of the semantic factor (*F*_(1,19)_ = 4.72, *p* = 0.03; $\eta_{\text{p}}^{\text{2}}$ = 0.04), with larger FN400 amplitude for semantically unrelated pairs than semantically related pairs.

#### LPC: Recollection

##### Old/new effect (Figure 5A)

We conducted paired *t*-tests for all conditions to estimate old-new effects: “related identical”, “related rearranged”, “unrelated identical”, “unrelated rearranged” vs. new pairs. Results indicated that the amplitude of all old conditions were greater than new pairs: “related identical” (*t*_(38)_ = 4.39; *p* < 0.0001), “related rearranged” (*t*_(38)_ = −3.81; *p* = 0.0005), “unrelated identical” (*t*_(38)_ = 2.5; *p* = 0.02) and “unrelated rearranged” (*t*_(38)_ = −2.3; *p* = 0.03). No significant difference was observed between the amplitude of “unrelated identical” and “unrelated rearranged” pairs (*p* = 0.89).

**Figure 5:**
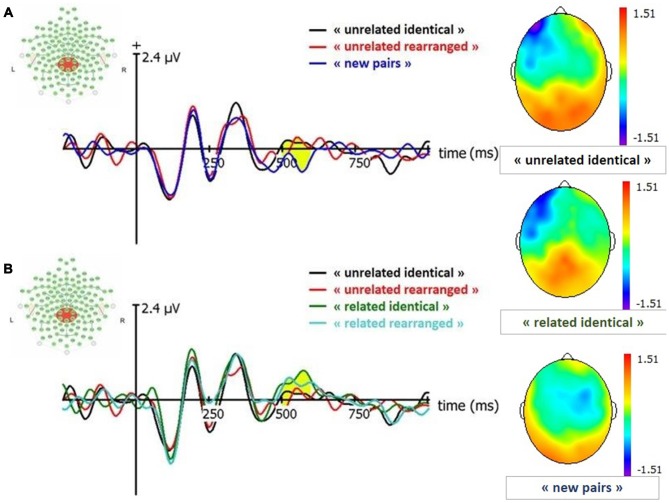
ERP and topographies for Late Positive Component (LPC; signal in CPz between 500–600 ms). “related identical”: semantically related identical pairs; “related rearranged”: semantically related rearranged pairs; “unrelated identical”: semantically unrelated identical pairs; “unrelated rearranged”: semantically unrelated rearranged pairs; “new pairs”: semantically unrelated new pairs. **(A)** Old/New effect for unrelated pairs. **(B)** Effect of semantic factor on Condition. Yellow shaded areas correspond to significant differences between conditions.

##### Effect of semantic relatedness (Figure 5B)

A 2 (condition: identical, rearranged) × 2 (semantic: related, unrelated) ANOVA conducted on the amplitude of the LPC in CPz revealed a significant main effect of the semantic factor (*F*_(1,19)_ = 7.38, *p* = 0.008; $\eta_{\text{p}}^{\text{2}}$ = 0.09) with semantically related pairs characterized by a larger LPC amplitude than semantically unrelated pairs.

##### Right parietal and occipital extension

We conducted paired *t*-tests for all conditions to estimate old-new effects: “related identical”, “related rearranged”, “unrelated identical”, “unrelated rearranged” vs. new pairs. Analyses indicated that in Oz the amplitude of “related identical” pairs was significantly greater than new pairs (*t*_(38)_ = 2.42; *p* = 0.02), and amplitude of “unrelated identical” in Oz was also greater than new pairs (*t*_(38)_ = 0.2.28; *p* = 0.03), and there was no significant difference for other conditions. In parietal areas, the amplitude of respectively “related identical” pairs and “related rearranged” was significantly greater than new pairs (related identical : *t*_(38)_ = 2.06; *p* = 0.05; related rearranged *t*_(38)_ = −2.28; *p* = 0.03). No other significant difference was found (*p* = 0.09 to *p* = 0.91).

### Debriefing

All participants noticed the presence of semantic categories, estimating the proportion of semantically related pairs at study to 44.3 ± 8.7% (the actual proportion was 50%). They spontaneously named 9 ± 2.3 semantic categories, and 17.3 ± 1.4 categories in response to cues provided by the experimenter. The semantic link between items was reported to be helpful by 75.7% of the participants at study, and at test was reported to be disadvantageous by 54.5%, as having made no difference by 54.5%, and to be helpful by 15.1% (several answers possible). Swapping the position of pictures at test was reported to have made no difference by 54.5%, was a hindrance for 36.3%, and reported as being helpful by 9.0% (several answers possible). These results suggest that semantic links facilitated response at study and therefore constituted an index of encoding.

## Discussion

The aim of this study was to identify the electrophysiological correlates of associative retrieval of pairs of pictures that are either strongly or weakly semantically related. The behavioral results showed no effect of semantic relatedness on accuracy, however, ERP revealed different patterns of associative recognition on the early mid-frontal and late parietal potentials. In the early mid-central component, we observed both a semantic effect (i.e., lower amplitude for semantically related compared to all other unrelated pairs) and a condition effect for the semantically unrelated pairs (i.e., an unexpectedly larger negativity for identical compared to rearranged and new pairs). Finally, we found a further late parietal old/new effect for both related and unrelated pairs, providing evidence that associative recognition of pairs of drawings relies on recollection, regardless of whether the associative link was semantic or not.

### Distinguishing Semantic from Item Specific Processing on Early Mid-Central Potential

The semantic effect on the early mid-central potential may reflect an automatic conceptual processing of similarities among semantically related pairs. First, debriefing revealed that all participants correctly identified the semantic associations, which could have guided both encoding and retrieval, according to encoding/retrieval match (Tulving and Thomson, 1973). Semantic cues are based on pre-existing intra-category associations between items (Wang and Morris, 2010), identified on the basis of their common perceptual and conceptual properties (Clark and Gronlund, 1996), and are automatically encoded during incidental learning of semantically related drawings (Hawco et al., 2013). Second, the early mid-frontal potential is thought to support both the familiarity FN400 potential during episodic retrieval, and semantic integration of related items (Bridger et al., 2012; Tibon et al., 2014b). The amplitude reduction found for all related pairs suggests particular retrieval operations, driven by a semantic processing of similarities (Voss and Federmeier, 2011), and would reflect the lower brain effort required to integrate related items into their semantic context (McPherson and Holcomb, 1999). We hypothesized a semantic effect, possibly due to a conceptual processing, but we cannot exclude another hypothesis that focuses on a familiarity-based recognition (i.e., unitiation) of related identical pairs (Zimmer and Ecker, 2010). Indeed familiarity processing is not usually associated with a FN400 effect (Wang et al., 2012).

Considering the condition effect for unrelated or weakly associated pairs (i.e., larger negativity for identical pairs), we hypothesize retrieval processes distinguishing identical from rearranged pairs, which differ by the type of associative information they contain. Larger negativity was first identified on the ERP literature as the reversed old/new effect (old more negative than new) in directed forgetting paradigms (Nowicka et al., 2009). Greater negativity on the early mid-central potential has recently been hypothesized to reflect the retrieval operation of the information associated with a pictorial cue (Tibon and Levy, 2014b), especially when the recall of the target fails (Tibon and Levy, 2014a). Similarly, we hypothesize that this larger early mid-central potential for unrelated target pairs could generate an additional retrieval effect focusing on cue processing, in the context of associative recognition. These processes may implicate perceptual properties as suggested by Langley (2010), who described larger FN400 when pictures encoding was oriented toward a perceptual processing opposed to a conceptual one.

### The Effect of Retrieval of Discriminant Associative Information on the LPC Potential

Accuracy was not improved by semantic relatedness contrary to our predictions. Each semantic category contained a large number of items (20), and some categories could have been perceived as one superordinate category, making the semantic cues less specific. Semantic facilitation would be observed with more distinctive semantic categories. Finally and as evidenced by the debriefing, common semantic properties of related pairs facilitated the study phase, i.e., required less cognitive resources but generated fewer contextual cue, which may have led to a lack of memory advantage at test.

Considering the LPC potential for both related and unrelated pairs, we found a brief (100 ms) LPC old/new effect. Recollection for pictures may reflect the actual matching of representations stored in memory with perceptual representations, and would be faster than words, in accordance with the distinctiveness account of the picture superiority effect (Ally and Budson, 2007), and since it yields more vivid representations than words (Tibon et al., 2014a). The occipital (Oz) extension observed in the present study and the right parietal tendency might be more specific to pictorial stimuli (Achim and Lepage, 2005).

We reported a condition effect both for related and unrelated pairs, confirming that associative recognition of picture pairs relies on recollection. We also highlighted a late semantic effect detected by the LPC potential, i.e., a greater LPC amplitude for semantically related pairs, contrary to our working hypothesis. First, this greater LPC may reflect an increased brain effort to achieve recollection (Rugg and Wilding, 2000), corresponding to an effortful associative information retrieval. This hypothesis is in accordance with behavioral results in favor of a higher associative discrimination index for unrelated compared to related pairs (i.e., more false alarms for the latest), in line with our predictions. High false recognitions for related pairs may be due to a possible confusion between items that shared the same semantic category (Poirier et al., 2012). Second, associative recognition could operate a “recall-to-reject” process, in which the participant actively remembers the target pairs for excluding rearranged ones, as identified with single or associative picture recognition (Xu and Malmberg, 2007), and associated with the LPC old/new effect (Curran and Cleary, 2003). The specific features and distinctiveness of unrelated items (Mäantylä, 1986) may have facilitated the “recall-to-reject” process, leading to a lower amplitude for unrelated pairs compared to related ones.

In conclusion, we have established that associative information guides encoding and retrieval strategies of pairs of pictures. We found that associative information requires recollection, regardless of the type of associative information, authenticated by an LPC old/new effect. The mid-parietal and right occipito-parietal topography of this effect may be specific to pictures. For semantically related pairs, retrieval requires searching for a semantic cue, i.e., similarities between items, which is reflected by a smaller amplitude of the early mid-frontal potential, followed by a larger LPC old/new effect both of which reflect the additional neural effort needed to achieve recollection. For unrelated pairs, we found an unexpected larger negativity on early mid-frontal potential, followed by a more limited late parietal old/new effect. This larger negativity argues in favor of additional mnemonic process, which may implicate the analysis of perceptual properties of picture pairs. Although the present study informs us about the different associative strategies used in memory, our paradigm did not allow us to investigate the electrophysiological correlates of perceptual processing that may operate in addition to semantic processing. Further research is needed to identify the contribution of dual-coding applied to pictures on the retrieval of associative memories.

## Author Contributions

PD contributed to the conception or design of the work, data collection, data analysis and interpretation, drafting the article, critical revision of the article and final approval of the version to be published. PC and FD contributed to data collection, data analysis and interpretation and final approval of the version to be published. AL, DMB, J-MB, PG and FE contributed to conception or design of the work, data analysis and interpretation, critical revision of the article and final approval of the version to be published. BG-G contributed to the conception or design of the work, data analysis and interpretation, drafting the article, critical revision of the article and final approval of the version to be published.

## Conflict of Interest Statement

The authors declare that the research was conducted in the absence of any commercial or financial relationships that could be construed as a potential conflict of interest.
