# Supplementary material for: Impact of Semantic Relatedness on Associative Memory: An ERP Study
Source: Front Hum Neurosci. 2017 Jun 29;11:335. doi: 10.3389/fnhum.2017.00335 (PMC5489662; doi:10.3389/fnhum.2017.00335)
Supplement: Supplementary file 1 [file Data_Sheet_1.docx]

**Supplementary data:**

**list of semantic categories**

Pets

Bathroom-related items

Sporting goods

Boats

Jewelry

Fruits

Insects

Musical instruments

Games

Vegetables

Wild mammals

Garden equipment

Electronic equipment

School-related items

Furniture

DIY tools

Kitchen utensils

Clothes

Motor vehicles

Meals and food

Table 1: percentage of hits and misses for “related identical” and “unrelated identical” pairs, and percentage of correct rejections and false alarms for “related rearranged”, “unrelated rearranged” and new pairs.

| IDENTICAL | Hits (sd) | Misses (sd) |
| --- | --- | --- |
| Semanticaly Related | 65.13 (7.93) | 34.88 (7.93) |
| Semanticaly Unrelated | 62.38 (11.02) | 37.63 (11.02) |
|  |  |  |
| REARRANGED | Corrects rejections (sd) | False alarms (sd) |
| Semanticaly Related | 63.50 (10.71) | 36.50 (10.71) |
| Semanticaly Unrelated | 76.25 (10.24) | 23.75 (10.24) |
| NEW | 97.50 (5.07) | 2.50 (5.07) |

Table 2: average number of artefact-free trials in each condition of interest (mean, standard-deviation, minimum, maximum).

|  | New pairs | “Unrelated identical “pairs | “Unrelated rearranged” pairs | “Related identical” pairs | “Related rearranged” pairs |
| --- | --- | --- | --- | --- | --- |
| Mean | 24,12 | 18,28 | 19,12 | 18,75 | 17,14 |
| Standard deviation | 4,97 | 2,69 | 2,69 | 2,37 | 3,13 |
| Minimum | 15 | 15 | 15 | 16 | 15 |
| Maximum | 30 | 22 | 23 | 22 | 24 |
